# Advancing equity in breast cancer care: strategies to enhance mammography screening among women with disabilities in Saudi Arabia

**DOI:** 10.3389/fonc.2025.1544468

**Published:** 2025-06-25

**Authors:** Huda I. Almohammed, Zuhal Y. Hamd, Mohamed M. Abuzaid

**Affiliations:** ^1^ Department of Radiological Sciences, College of Health and Rehabilitation Sciences, Princess Nourah bint Abdulrahman University, Riyadh, Saudi Arabia; ^2^ Medical Diagnostic Imaging Department, College of Health Sciences, University of Sharjah, Sharjah, United Arab Emirates; ^3^ Research Institute for Medical and Health Sciences, University of Sharjah, Sharjah, United Arab Emirates

**Keywords:** disabilities, breast cancer, women, Saudi Arabia, mammography, screening

## Abstract

**Introduction:**

Breast cancer is a significant public health issue worldwide, especially in Saudi Arabia, where it is the most prevalent cancer among women. Early detection through mammography is crucial, but disparities in screening accessibility for women with disabilities are evident. This study builds upon previous research to address these disparities. The study aims to develop and evaluate comprehensive educational and healthcare strategies to enhance mammography screening participation among Saudi women with disabilities. The study focuses on increasing screening rates and healthcare equity by addressing barriers and leveraging facilitators specific to this demographic.

**Methods:**

This cross-sectional study was conducted across various regions in Saudi Arabia, targeting women with disabilities aged 40–69 years. Data collection involved a structured questionnaire, distributed both physically and online, focusing on demographic details, breast cancer screening practices, and barriers to access. The study also examined the effectiveness of various educational and healthcare interventions. Data analysis utilized IBM SPSS Statistics Version 26, employing chi-square tests and logistic regression.

**Results:**

The study’s demographic analysis revealed a diverse population with various disabilities and educational levels. Findings showed that 54.4% of participants found healthcare facilities only partially accessible, and 62.5% faced barriers in accessing breast cancer screening services. The majority (91.5%) had support systems or caregivers. Interventions focusing on educational campaigns, healthcare provider training, and improving facility accessibility were analyzed for their impact.

**Conclusion:**

The study underscores the necessity of inclusive healthcare practices and highlights the effectiveness of targeted interventions in improving mammography screening rates among women with disabilities. Collaborative efforts among healthcare providers, policymakers, and advocacy groups are crucial for addressing these disparities. Future research should focus on understanding specific challenges across diverse populations, assessing the long-term impact of improved screening rates, and evaluating the cost-effectiveness of interventions. This research contributes significantly to advancing healthcare equity in breast cancer screening for women with disabilities in Saudi Arabia.

## Introduction

Breast cancer remains a major public health concern globally, with its early detection critically dependent on effective screening practices, particularly mammography. In Saudi Arabia, breast cancer is the most common cancer among women, accounting for a significant portion of female cancer cases. The importance of mammography as a screening tool cannot be overstated, as it plays a pivotal role in the early detection and management of breast cancer. However, there exists a notable disparity in the accessibility and utilization of mammography screening, especially among women with disabilities. This disparity is not only a matter of concern in terms of healthcare equity but also reflects broader societal and systemic issues that need addressing 1.

The first part of this research, titled “Breaking Barriers: Improving Mammography Screening Accessibility and Quality of Care for Breast Cancer Women with Disabilities in Saudi Arabia,” 2. laid the groundwork by highlighting the significant challenges and disparities faced by Saudi women with disabilities in accessing mammography screening services. It revealed that a majority of the participants had irregular screenings, lacked tailored information on breast cancer screening, and faced several psychological and logistical barriers. These barriers include physical limitations, lack of knowledge, fear and embarrassment, anxiety about the examination process, dependency on others, and inadequate understanding of healthcare professionals about their disability 3. These findings underscore the critical importance of tailored interventions and comprehensive education to bridge the gap in screening rates and ensure equitable access to essential healthcare services for these populations 4.

In light of these findings, this second part of the research aims to build upon the initial study by developing and assessing the effectiveness of targeted educational and healthcare strategies to improve mammography screening participation among women with disabilities in Saudi Arabia. This objective is pursued in recognition of the fact that women with disabilities are an often overlooked and underserved demographic in breast cancer screening initiatives. The research seeks to explore the barriers and facilitators influencing breast cancer screening participation, focusing on the diverse needs of women with different types of disabilities.

Saudi Arabia has witnessed significant advancements in healthcare over the past few decades. However, women with disabilities often experience a double burden of gender and disability, which can lead to their health needs, particularly in areas such as breast cancer screening, being inadequately addressed. This issue is further complicated by cultural and social dynamics, which can influence perceptions and accessibility to healthcare services. The current study recognizes that addressing these challenges requires a multi-faceted approach, encompassing not only healthcare policy and practice but also broader societal attitudes and awareness.

The significance of this research lies in its focus on a group that is frequently marginalized in health discussions and policy-making. Women with disabilities face unique challenges in accessing healthcare services, and these challenges can be exacerbated in the context of breast cancer screening. The physical layout of screening facilities, the equipment used, and even the attitudes and knowledge of healthcare providers can all act as barriers to effective screening. Moreover, the psychological impact of disability, coupled with the stigma and misconceptions surrounding both disability and breast cancer, can further hinder access to screening 5.

In addition to addressing these practical and psychological barriers, the research also considers the role of information dissemination and education in improving screening rates among women with disabilities. The first part of the study highlighted a significant gap in awareness and knowledge about breast cancer and mammography among this group. This second part seeks to address this gap by developing targeted information and education campaigns that are accessible and relevant to women with disabilities. These campaigns are designed not only to inform and educate but also to empower women, enabling them to make informed decisions about their health and to advocate for their needs within the healthcare system 6.

Furthermore, this study also aims to explore the role of social support in enhancing screening participation. Support groups, healthcare professionals, and online resources have been identified as key sources of information and support for women with disabilities. By understanding the dynamics and impact of these support systems, the research seeks to develop strategies that leverage these resources to improve screening rates.

Breast cancer is the most frequently diagnosed malignancy among women in Saudi Arabia, with a significant increase in incidence over recent decades. The age-standardized incidence rate (ASR) rose from 15.4 per 100,000 in 1990 to 46.0 in 2021, with the Eastern region reporting the highest ASR at 52.2 per 100,000. Mortality rates also increased, with the ASR for deaths rising from 6.73 in 1990 to 9.77 in 2021, particularly affecting women aged 40–49 years. This upward trend underscores the need for early detection, accessible screening services, and inclusive public health strategies targeting vulnerable populations, including women with disabilities 7.

In conclusion, this research is an essential step towards improving healthcare equity in Saudi Arabia. By focusing on the specific needs and challenges faced by women with disabilities in accessing mammography screening, the study aims to develop comprehensive strategies that not only address these barriers but also promote a more inclusive and equitable approach to healthcare. The ultimate goal is to ensure that all women, regardless of their physical abilities, have access to the critical healthcare services they need for the early detection and effective management of breast cancer.

## Material and methods

### Study design and setting

A cross-sectional study design was adopted, extending from the initial research. The study was conducted across various regions in Saudi Arabia, encompassing urban and rural areas, to ensure a representative sample of the target population. The research was carried out over six months, from May to October 2024.

### Participants

The study included Saudi women with disabilities who were eligible for breast cancer screening according to the national guidelines. The inclusion criteria were: Saudi nationality, female, aged 40–69 years, identified as having a disability (physical, sensory, intellectual, or neuro-disabilities), and ability to provide informed consent. Exclusion criteria included women who were unable to complete the questionnaire independently and had no relative or caregiver to assist, those with a prior diagnosis of breast cancer, those currently undergoing treatment for any malignancy, and those who had participated in similar breast cancer awareness interventions within the previous six months. These exclusions were applied to minimize bias and ensure the accuracy of the findings regarding baseline knowledge and screening behaviors.

### Sample size calculation

The required sample size was calculated using Cochran’s formula for proportions with a 95% confidence level and a 5% margin of error, yielding an initial sample size of 385 participants. Given the estimated population of Saudi women with disabilities aged 40–69 is approximately, a finite population correction was applied where applicable. Despite this, 307 valid responses were collected. Although slightly below the ideal threshold, this sample size remains sufficient for exploratory and associative analysis, with acknowledged limitations addressed in the discussion.

### Data collection

Data was collected using a structured questionnaire based on insights from the previous studies. The questionnaire was translated into Arabic and validated by two professional translators to ensure accuracy and cultural relevance. It was distributed physically and online, facilitated by research assistants proficient in Arabic and English, to ensure wider reach and accessibility.

### Research instrument

The survey instrument included sections on demographic details (age, marital status, education level, type of disability, and duration of disability), breast cancer screening practices (frequency and type of screening), knowledge and awareness about mammography, and barriers to accessing screening services. Additionally, the questionnaire assessed the impact of various educational and healthcare strategies on the screening behaviors of the participants.

### Data analysis

Data analysis was performed using IBM SPSS Statistics Version 26. Quantitative variables were presented as frequencies and percentages. Chi-square tests were used to compare categorical variables, and logistic regression was employed to identify factors influencing mammography screening behaviors. The analysis focused on evaluating the effectiveness of interventions in improving mammography screening rates and understanding the barriers and facilitators affecting screening participation.

### Educational and healthcare interventions

Based on the findings of the previous studies, several interventions were introduced. These included:

1. Targeted Education Campaigns: Tailored educational materials were developed and disseminated, focusing on the importance of breast cancer screening, specifically designed for women with different types of disabilities.
2. Healthcare Provider Training: Training sessions for healthcare providers were conducted to enhance their understanding of the unique needs of women with disabilities and to improve communication and screening practices.
3. Accessibility Improvements: Efforts were made to enhance the physical accessibility of mammography screening facilities and to provide adaptive equipment where necessary.
4. Community Engagement: Collaboration with disability organizations and support groups to promote awareness and encourage participation in breast cancer screening.

### Ethical considerations

Ethical approval for this study was obtained from the Institutional Review Board (IRB) at Princess Nourah bint Abdulrahman University, Riyadh City, KSA (IRB Log Number: 24-0001). All participants provided informed consent, and confidentiality was maintained throughout the study.

## Results

### Demographic distribution

**Table 1** outlines the study participants’ demographic distribution. Regarding marital status, a minority of the participants are married (67 individuals, 21.8%), while a substantial majority are unmarried (240 individuals, 78.2%). Regarding educational attainment, a small fraction reported no formal education (6 individuals, 2%), intermediate education written by 88 individuals (28.7%), secondary education by 57 individuals (18.6%), and a Bachelor of Science (B.Sc.) degree is the highest reported level of education for a majority of the participants, with 156 individuals (50.8%) having attained this level. The mean age of the participants is approximately 47.93 and the standard deviation is about 6.15.

**Table 1:** Participant demographics: marital status and educational levels.

Variable	Category	n	%
Marital status	Married	67	21.8
	Unmarried	240	78.2
Level of education	No	6	2
	Intermediate	88	28.7
	Secondary	57	18.6
	B.Sc.	156	50.8

### Participant disabilities and duration of condition

**Table 2** details the study participants’ disabilities, conditions, and disability duration. Learning and physical disabilities are the most common, reported by 35.2% and 35.8% of participants, respectively. 8.8%, 7.8%, and 12.4% of participants say intellectual, neuro, and sensory disabilities.

**Table 2:** Breakdown of participant disabilities and duration of condition.

Variable	Category	n	%
Type of disability	Intellectual	27	8.8
	Learning	108	35.2
	Neuro	24	7.8
	Physical	110	35.8
	Sensory	38	12.4
What type of disability do you have	Intellectual -Down	14	4.6
	Intellectual	13	4.2
	Learning - dyslexia	3	1
	Learning -ADHD	105	34.2
	Neuro -MS	24	7.8
	Physical	110	35.8
	Sensory (blindness)	11	3.6
	Sensory (deafness)	27	8.8
How long have you been living with your disability?	Since birth	199	64.8
	Less than 10 years	15	5.0
	10–19 years	71	23.3
	More than 20years	22	7.1

The most common learning disability is Attention Deficit Hyperactivity Disorder (ADHD), affecting 34.2% of participants. General physical disabilities affect 35.8% of participants. Down syndrome is reported by 4.6% of participants, and other intellectual disabilities by 4.2%. Blindness accounts for 3.6% of sensory disabilities, while deafness accounts for 8.8%. Neuro disabilities include Multiple Sclerosis (MS), which affects 7.8% of participants.

Most participants (64.8%) have had their disability since birth. 23.3% of the population has lived with a disability for 10–19 years, while 5.0% and 7.1% have lived with it for less than 10 and 20 years, respectively.

This distribution shows the diversity of disabilities in the study’s demographic and the different needs to be considered when providing healthcare services like mammography screening.

### Healthcare accessibility and caregiver assistance for women with disabilities in mammography screening

The following data present information on healthcare accessibility for female patients with disabilities regarding mammography screening.

#### Healthcare accessibility

**Table 3** addresses the accessibility of healthcare facilities. A small percentage of participants (8.1%) report that healthcare facilities in their area are not accessible at all for individuals with disabilities, while over half (54.4%) find them only partially accessible. However, a significant portion (37.5%) states that healthcare facilities are accessible.

**Table 3:** Healthcare accessibility for women with disabilities.

Variable	Category	n	%
Are healthcare facilities in your area accessible for individuals with disabilities?	No, not at all	25	8.1
	Partially	167	54.4
	Yes	115	37.5
Have you faced any barriers or challenges while accessing breast cancer screening services due to your disability?	No	115	37.5
	Yes	192	62.5
Do you have a support system or caregiver who helps you with breast cancer screening and protection?	No	26	8.5
	Yes	281	91.5
Mammogram facilities accommodating and accessible for your disability?	Partially	132	43
	Yes, completely	175	57

When it comes to barriers or challenges faced while accessing breast cancer screening services, a substantial majority of participants (62.5%) have encountered difficulties due to their disability, whereas 37.5% have not faced any barriers.

In terms of support, an overwhelming majority of participants (91.5%) report having a support system or caregiver to assist with breast cancer screening and protection. Furthermore, when asked if mammogram facilities are accommodating and accessible for their disability, 57% of participants affirm complete accommodation and accessibility, while 43% only partially agree.

#### Assistance from caregiver

**Figure 1:**
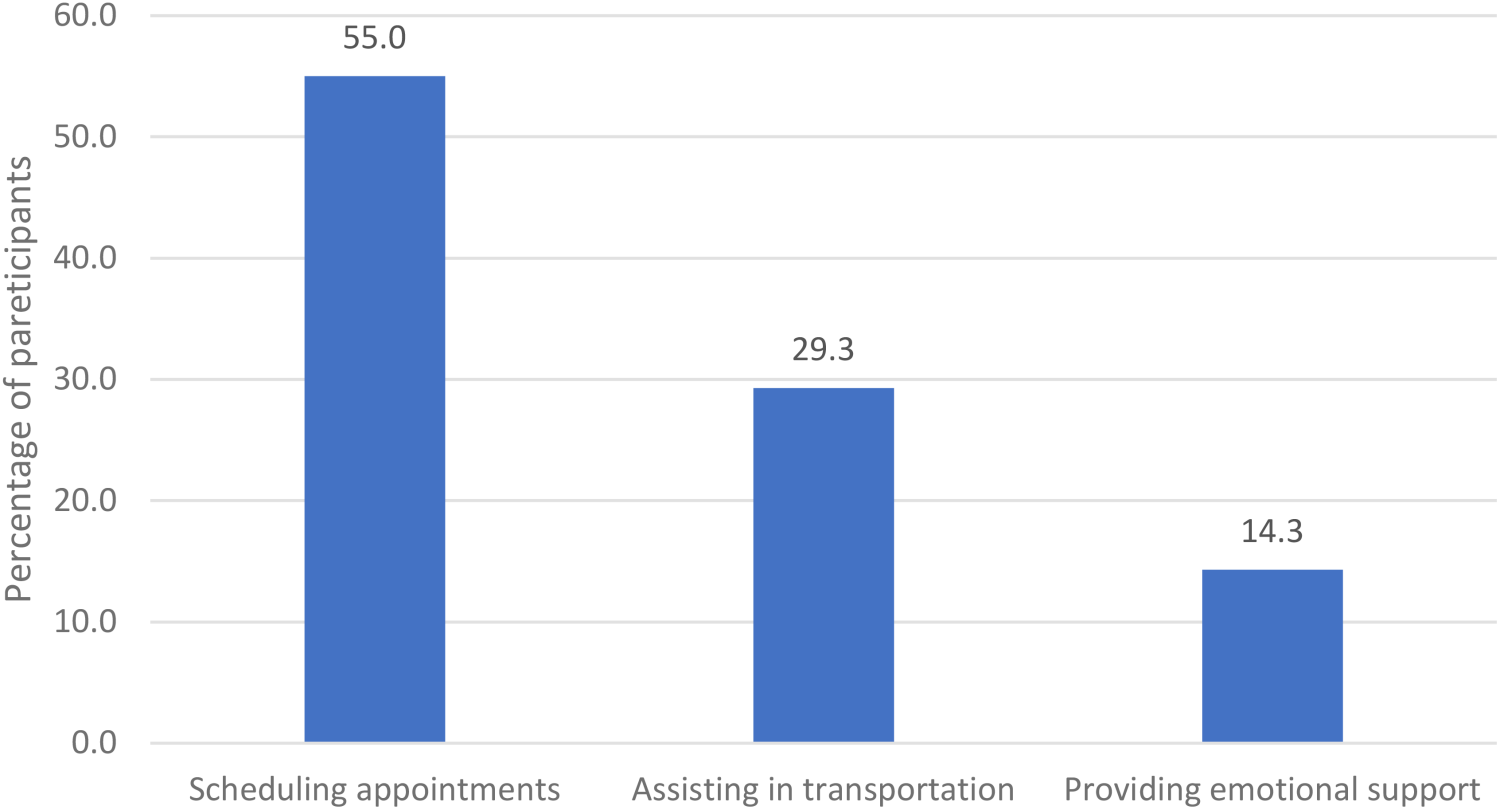
Types of assistance received from caregivers.

**Figure 1** focuses on the type of assistance provided by caregivers. Scheduling appointments is the most common service form, with 55% of participants indicating this type of support. Transportation assistance is also notable, with 29.3% of participants requiring it. Emotional support and communicating with healthcare workers on behalf of the patient are less frequently needed but still significant, highlighted by 14.3% of participants for emotional support.

**Figure 1** highlights the critical areas where interventions can be implemented to improve accessibility and support for female patients with disabilities in mammography screening.

### Impact of disability type on accessibility and accommodation in mammography screening: a chi-square analysis

Chi-square analysis of mammography screening accessibility for women with disabilities indicates significant variations in service accessibility and quality based on disability type. Patients with neurological and physical conditions reported better facility accommodations than those with intellectual, learning, or sensory challenges, as reflected by a chi-square value of X² (8, N = 307) = 160.90, p < 0.000. This demonstrates a substantial association between disability type and healthcare access.

Barriers during screening are also significantly linked to disability type, with a chi-square value of X² (4, N = 307) = 61.125, p < 0.000, suggesting that specific disabilities are associated with more screening challenges.

Support from caregivers shows considerable variability by disability type (X² (4, N = 307) = 54.418, p < 0.000), impacting the ability to access screening services. Moreover, the chi-square test on facility accommodation (X² (4, N = 307) = 35.952, p < 0.000) indicates that neurological and physical conditions are more likely to be fully accommodated.

These results underscore the need for tailored healthcare facility design and service provision approaches to ensure equitable mammography screening access for all disability categories.

## Discussion

The current study’s findings, which show significant disabled women’s mammography screening accessibility disparities, support the literature on disability’s intersectionality with other demographic factors, such as race/ethnicity, and its effects on breast cancer care.

Balogun et al. (8) highlight the challenges African American and Hispanic women with disabilities face in timely post-abnormal mammography follow-up. According to our study, disability type affects access and accommodations. Targeted interventions for layered disparities, especially for minority women with disabilities, are needed. McCarthy et al. (9) note disability-related breast cancer treatment and survival disparities, which accords with our findings on mammography screening accessibility. Their call for better access and support services parallels our study’s call for comprehensive strategies to improve care for all disabled women.

Women with disabilities may need tailored communication and accommodation strategies to participate in their healthcare decisions fully. However, the American Cancer Society’s guidelines (10) emphasize regular screenings and shared decision-making. We agree with Iezzoni and Long-Bellil (11) that women with disabilities need continuous and comprehensive preventive care beyond the annual exam. Our study’s focus on accessibility is relevant to their focus on overcoming barriers to preventive services, including mammography. Disparities must be addressed systemically, as shown by research on reducing healthcare provider biases (12) and mammography trends for women with disabilities (13). Facility accommodations and caregiver support are crucial to equitable breast cancer screening, as shown by our study.

The literature emphasizes the need for a nuanced approach to address the multifaceted nature of breast cancer disparities for women with disabilities. Our study highlights key intervention areas, including healthcare facility accessibility, caregiver support, and equitable care for women with diverse disabilities.

This study’s findings on disabled women’s mammography screening accessibility complement health literacy and education literature. These studies show that tailored health information and accessible resources improve breast cancer screening awareness and decision-making for disabled women. Burgess et al. (14) show how provider biases affect screening and treatment decisions. This paper emphasizes the importance of educational interventions to reduce provider biases and promote equitable care. Based on our findings, this means ensuring that health information and services are delivered without bias, especially toward women with disabilities. McCarthy et al. (9) highlight the treatment and survival disparities for women with disabilities, highlighting the need for comprehensive care that addresses their unique challenges. Our study found that support systems, including caregiver assistance, help people access screening services. Mitby et al. (15) emphasize the need for specialized education for childhood cancer survivors to reduce disparities. This study focuses on a different population, but tailored educational support applies to ours. It suggests that health literacy interventions for disabled women may increase their breast cancer screening participation. Our study shows the need for accessible mammography screening and the importance of educating women with disabilities to make informed decisions and engage with preventive health services.

The current study’s insights into enhancing mammography screening accessibility and accommodation for women with disabilities align with the broader literature advocating for systemic strategies to improve equity in breast cancer care.

Burgess et al. (14) underscore the impact of provider biases on healthcare disparities, which extends to the domain of breast cancer screening. Their conclusions suggest that provider training should include modules to counteract stereotypes and biases, ensuring that all women, irrespective of race, ethnicity, or disability, receive equitable care.

McCarthy et al. (9) discuss the disparities in treatment and survival rates for women with disabilities, advocating for targeted interventions to improve access and outcomes. This reinforces our study’s call for healthcare systems to ensure that women with disabilities are not disadvantaged by a lack of appropriate accommodations during screening.

Institute of Medicine (US) Committee on Health Literacy et al. (16) focus on health literacy, a fundamental aspect that can empower women with disabilities to participate actively in their healthcare. Enhancing health literacy is a pivotal strategy that our study also implies, as it is integral to informed decision-making and optimizing breast cancer screening services utilization.

Collectively, these studies affirm the need for multifaceted strategies to improve accessibility and accommodation in breast cancer care for women with disabilities. These include comprehensive provider training, investment in accessible imaging technology, and health literacy and communication improvements. Our study contributes to this discourse by demonstrating the practical implications of such strategies and emphasizing the role of healthcare systems in their implementation to ensure that women with disabilities receive equitable care.

## Conclusions

The collective research on breast cancer care equity and mammography screening for women with disabilities underscores an imperative: healthcare access barriers must be dismantled. This body of work confirms that inclusive healthcare is a fundamental right for all women, irrespective of disability status, and delineates several strategies to enhance screening accessibility.

Studies demonstrate that enhancing the physical infrastructure of mammography centers, developing patient education materials tailored to diverse needs, and equipping providers with the skills to communicate effectively with women with disabilities are pivotal measures. These initiatives are essential to ensure equitable access to breast cancer screening services and mitigate health outcomes disparities.

The literature further stresses the critical collaboration between healthcare professionals, policymakers, and advocacy groups in eradicating impediments to breast cancer screening for women with disabilities. There is a call for research-informed policy reforms and shifts towards more inclusive healthcare systems that are fundamental to crafting and enacting comprehensive programs designed for women with disabilities.

While the extant research provides a substantial platform, it also identifies areas ripe for further exploration. A more profound comprehension of the specific challenges faced by women with disabilities, particularly in varied cultural and economic milieus, is essential. Such insights are anticipated to refine intervention strategies to meet the needs of this population more effectively.

Additionally, there is a need for longitudinal studies to assess the impact of increased screening on breast cancer diagnosis, treatment, and survival rates among women with disabilities. Insights from these studies are anticipated to bolster the impetus for prioritizing equity in breast cancer care for this often overlooked demographic. Moreover, future research ought to evaluate the cost-effectiveness and sustainability of the implemented interventions. This information is vital for guiding healthcare policymakers and entities in resource allocation and strategic planning to enhance mammography screening accessibility for women with disabilities.

In essence, the literature calls for a commitment to inclusive healthcare and the adoption of specific interventions to uplift mammography screening rates among women with disabilities. It advocates for a synergistic approach involving policy innovation and ongoing research to realize enduring and comprehensive improvements in breast cancer screening and care, thus advancing the overarching goal of healthcare equity.

## Recommendation

The study recommends a multipronged approach to improve mammography screening for women with disabilities in Saudi Arabia. Firstly, healthcare facilities should enhance physical accessibility and adapt screening equipment to cater to various disabilities. Training healthcare providers in disability awareness and communication is crucial for sensitive and effective patient interaction. Implementing comprehensive educational campaigns tailored to women with disabilities will improve awareness and encourage participation in screening programs. Additionally, fostering collaborations between healthcare institutions, policymakers, and disability advocacy groups is vital for sustained improvements. These recommendations aim to bridge the gap in breast cancer screening accessibility and promote equity in healthcare services for women with disabilities.

## Data Availability

The original contributions presented in the study are included in the article/supplementary material. Further inquiries can be directed to the corresponding author.
